# Long-term follow-up on HIV infected and non-infected women with cervical cancer from Tanzania: staging, access to cancer-directed therapies and associated survival in a real-life remote setting

**DOI:** 10.1186/s12885-022-09966-7

**Published:** 2022-08-15

**Authors:** Laura Glasmeyer, Ruby Doryn Mcharo, Liset Torres, Tessa Lennemann, Elizabeth Danstan, Nice Mwinuka, Mona Judick, William Mueller, Wilbert Mbuya, Michael Hölscher, Ralph Lellé, Christof Geldmacher, Arne Kroidl, John Rwegoshora France

**Affiliations:** 1 Division of Infectious Diseases and Tropical Medicine, Medical Center of the University of Munich (LMU), Munich, Germany; 2 Charité-Universitätsmedizin Berlin, Berlin, Germany; 3 Mbeya Medical Research Center (MMRC), National Institute for Medical Research (NIMR), Mbeya, Tanzania; 4 Department of Obstetrics and Gynaecology, Mbeya Zonal Referral Hospital, Mbeya, Tanzania; 5 German Center for Infection Research (DZIF), partner site, Munich, Germany; 6 Division of Gynecology, University of Muenster, Muenster, Germany

**Keywords:** Cervical cancer, HIV, Cancer-directed therapy (CDT), FIGO staging, Mortality, Cancer screening, Tanzania

## Abstract

**Background:**

Worldwide 85% of cervical cancer (CC) related deaths occur in low- and middle-income countries. Sub-Saharan Africa is burdend by an overlapping high incidence of CC as well as HIV infection, a risk factor for HPV associated disease progression. Recent upscaling of CC screening activities increased the number of CC diagnoses in a previous unscreened population. The aim of the 2H study was to follow up on women with CC in the context of available health care services in Tanzania in relation to their HIV infection status.

**Methods:**

This longitudinal observational cohort study included women with histological confirmed CC from Mbeya, Tanzania, between 2013–2019. All women were referred for CC staging and cancer-directed therapies (CDT), including surgery and/or radio-chemotherapy, or palliative care. Annual follow-up focused on successful linkage to CDT, interventions and survival. We assessed factors on compliance, used Kaplan–Meier-Survivor functions to evaluate survival time and poisson regression models to calculate incidence rate ratios on mortality (IRR) two years after diagnosis.

**Results:**

Overall, 270 women with CC (123 HIV infected) were included. Staging information, available in 185 cases, showed 84.9% presented with advanced stage disease (FIGO ≥ IIB), no difference was seen in respect to HIV status. HIV-infected women were 12 years younger at the time of cancer diagnosis (median age 44.8 versus 56.4 years, *p* < 0.001). Median follow up period was 11.9 months (range 0.2–67.2). Survival information, available in 231 cases, demonstrated for women diagnosed in early-stage disease a median survival time of 38.3 months, in advanced-stage 16.0 months and late-stage disease 6.5 months after diagnosis. Of all women, 42% received CDT or palliative support. HIV co-infection and education were associated with higher health care compliance. CDT was significantly associated with lower 2-year mortality rates (IRR 0.62, *p* = 0.004). HIV coinfection did not impact mortality rates after diagnosis.

**Conclusion:**

High numbers of advanced and late staged CC were diagnosed, compliance to CDT was low. A beneficial impact of CDT on CC mortality could be demonstrated for local health care services. This study indicates challenges for successful linkage and supports an effective scale up of cancer care and treatment facilities.

## Background

One in twenty-four women from low-income countries develops cervical cancer (CC) until the age of 79 years [1]. Worldwide more than 85% of all CC related deaths occur in low- and middle-income countries (LMICs) [2, 3]. Tanzanian women in particular are globally among the most affected women by this cancer with an age standardized incidence rate of 62.5 per 100,000 women as compared to 13.3 in the world and with an age standardized mortality rate of 42.7 per 100,000 women as compared to 7.3 in the world [2, 4]. The vast majority of cervical malignancies are caused by High-Risk Human Papilloma Viruses (HR-HPV) infection [5]. Human Immunodeficiency Virus (HIV) coinfected women have a five- to ninefold increased risk for disease progression from HPV infection to CC [6–9]. Due to an overlapping high burden of CC and HIV infection in sub-Saharan Africa (SSA), six countries, namely Tanzania, Malawi, Mozambique, South Africa, Uganda and Zimbabwe, account for about 50% of all HIV infected women with CC worldwide [9].

Studies from 2009–2019 have suggested a higher risk of death during the first year following cancer diagnosis for HIV infected patients in Botswana, Uganda and northern Tanzania [10–13]. The introduction of antiretroviral therapy (ART) reduced overall mortality from AIDS. Lower rates of cervical lesion incidence and progression and higher rates of regression after HR-HPV infection were seen in HIV infected women treated with ART, but with comparatively little effect on mortality after CC diagnosis [10, 14].

Cervical screening programmes to detect and treat precancerous lesions, and HPV vaccination programmes are the corner stones to reduce CC mortality in developing countries [3, 15]. Major scale outs in screening and first vaccination programmes are ongoing in many African countries. Tanzania has progressed over the last decade in the fight against CC by implementing a national screening programme in 2008, a single-visit, test and treat approach has been implemented since 2011 and the capacities have been scaled up country-wide over the years [16, 17]. According to the World Health Organization (WHO), women aged 30–49 should be prioritized to receive screening procedures; and sexually active HIV infected women should receive CC screening regardless of age [3, 18]. However, with up-scaling screening activities not only precancerous lesions are being diagnosed. In a previously unscreened population CC diagnosis is increasing as well, resulting in more patients in need for staging and CDT. To establish a comprehensive CC prevention programme, it is thus necessary to also provide tertiary prevention including access to cancer diagnostics, treatment and palliative care options [3]. The Mbeya region is located in the south of Tanzania and has one of the highest numbers in HIV prevalence within the country (13% in 2005, national adult prevalence 6.5%) [19]. It is home to 1,9 million people. One gynecologist covers the performance of CC staging procedures, offers guidance for further treatment options, and performs surgical interventions in this region. In the whole country, access to radio-chemotherapy has been limited to the only specialized cancer treatment center, the Ocean Road Cancer Institute (ORCI) in Dar Es Salaam. To plan further interventions, identify current problems in local patient-oriented care and public health several observational studies are needed to assess the diverse status quo of the current global health care situation for women with cervical cancer diagnosis, especially in more rural environments.

From 2013 until end 2020 we conducted the 2H (HIV and HPV) Study in Mbeya, Tanzania, evaluating the impact of HIV on HPV-associated disease, and specifically in this study to follow up on woman with CC diagnosis in the context of available health care services in Tanzania.

## Methods

### Study design and populations

The 2H study was a prospective, longitudinal cohort study that included HIV infected and non-infected women, who underwent cervical screening procedures in Mbeya, Tanzania. In this study we focused on women who were diagnosed with CC with the aim to analyze survival in the context of HIV co-infection and other risk-factors and to evaluate the uptake and impact of cancer care and treatment interventions, including compliance to offered health care services. The study was conducted at the National Institute for Medical Research-Mbeya Medical Research Center (NIMR-MMRC) in collaboration with the HIV Care and Treatment Clinic (CTC) and the META Gynecological Hospital of the Mbeya Zonal Referral Hospital (MZRH) in Mbeya. The MZRH (https://mzrh.go.tz/index.html) is one of four tertiary hospitals within the country and provides health care services to the south-west of Tanzania, serving a mainly rural or semi-rural population. Ethical approval was obtained from the Mbeya Medical Research and Ethics review Committee, the Tanzanian National Health Research Ethics Committee, and the Ethics Committee of the University Munich (LMU).

Women aged 18 years and above, who attended the national cervical cancer screening programme, were included into this study. Exclusion criteria were prisoners, mentally disturbed women, or women in a serious health condition for whom study participation or informed consent procedures would imply an undue burden. Participant oral and written study information was provided by trained study nurses, written or for illiterate women thump printed informed consent in the presence of an impartial witness was required.

### Study procedures

Cervical cancer screening was performed according to the national guideline by trained and certified health care personnel. Screening included gynecological examination, speculum examination of the naive vagina and cervix uteri, and Visual Inspection using Acetic acid (VIA) on to the cervix uteri to identify aceto-white lesions suspicious for cervical intraepithelial dysplasia. In addition, samples for cytology and HPV-genotyping were taken from the ecto- and endocervix using an ayre’s spatula and a cytobrush, the results have been recently published [20]. In case of clinical cancer suspicion, biopsies were collected. All cyto-histological diagnostics were performed at the Pathology Department and Laboratory of the MZRH. Cytology results were categorized following the Bethesda classification system [21], and in case of High-grade Squamous Intraepithelial Lesions (HSIL) women were contacted to undergo biopsies and histological diagnostics for confirmation. Histology findings were reported as presence of normal mucosa, precancerous lesion grades as Cervical Intraepithelial Neoplasia (CIN) grade 1, 2, 3 or Adenocarcinoma in situ, or confirmation of invasive cervical cancer, either as Squamous Cell Carcinoma (SCC), Adenocarcinoma, or Adenosquamous Cell Carcinoma. Women diagnosed with CC were referred to the META Gynecological Hospital of the MZRH, where FIGO staging (International Federation of Gynecology and Obstetrics) according to the 2009 classification was performed [22]. Assessments for further medical procedures were performed by a gynecologist, including physical examination and pre-surgical abdominal and pelvic ultrasound scanning. Where indicated, radical hysterectomy according to Piver III was performed at the MZRH-Gynecology [23]. Suspicious lymph nodes were sent to pathology for determination of tumor involvement. Where surgical interventions were not recommended a referral for radio-chemotherapy to the Ocean Road Cancer Institute (ORCI) in Dar Es Salaam was initiated. The indication for cancer-directed therapy (CDT) or consideration of palliative care only was a shared decision between the patient and the META gynecologist at MZRH in harmonization with ORCI as applicable.

For all women with unknown HIV status, HIV testing was offered at baseline and during annual follow-up visits according to the national Voluntary Counselling and Testing (VCT) HIV test algorithm: a screening rapid test (Determine HIV1/2, Abbott Laboratories, South Africa), and if reactive, followed by a second confirmatory rapid test (Uni-Gold HIV Rapid Test, Trinity Biotech, South Africa). In the case of discordant results, an enzyme-linked immunosorbent assay (ELISA) (Bio-Rad GS HIV-1/HIV-2 PLUS O EIA, Bio-Rad laboratories GmbH, Germany) was performed. All tests were performed at the College of American Pathologists (CAP) accredited NIMR-MMRC laboratories. For HIV infected women HIV history and antiretroviral therapy (ART) information were collected through interviews and patient’s hospital charts. CD4 T-cell counts were analyzed from peripheral blood samples (Becton Dickinson FACSCount™ system).

Annual follow-up visits were targeted, which included for CC diagnosed women updated information on their general health status, quality of life, access to health care services, therapeutic interventions, and palliative care information. Participants who missed their follow-up date were reminded via telephone call or text message. Following three unsuccessful attempts, physical home tracing was conducted. Reasons and information related to early termination such as death, study withdrawal or relocation were recorded, women who could not be traced were classified as lost to follow-up (LTFU).

### Outcome measures and statistical analysis

Clinical and laboratory data were recorded in study specific case report forms and entered twice into the study tailored database which was programmed in Structured Query Language (SQL). Descriptive analyses reported the median and range for continuous variables, as well as the number and percentage of participants for binary and categorical variables, using Fisher’s exact test for binary and Mann–Whitney-U-Test in groups without standard distribution for continuous variables. To estimate relationships of different factors with health care compliance, we used a robust binary regression model to calculate risk ratios (RR) and performed a Wald test for hypothesis testing. For survival time analysis we used the Kaplan–Meier survivor function and a log-rank-test for assessment of significance. For binary regression analysis in mortality data, we used a robust Poisson regression model to calculate incidence rate ratios (IRR) two years after diagnosis. To test for dispersion, a chi-square goodness of fit test was performed. All reported p-values were two-sided and for all statistical tests an alpha level of < 0.05 was used to define significance. All statistical analyses were performed using Stata statistics software (version 16, StataCorp, College Station, TX, USA), graphs were additionally drawn in MS Excel and GraphPad Prism (version 9.0.0).

## Results

**Fig. 1:**
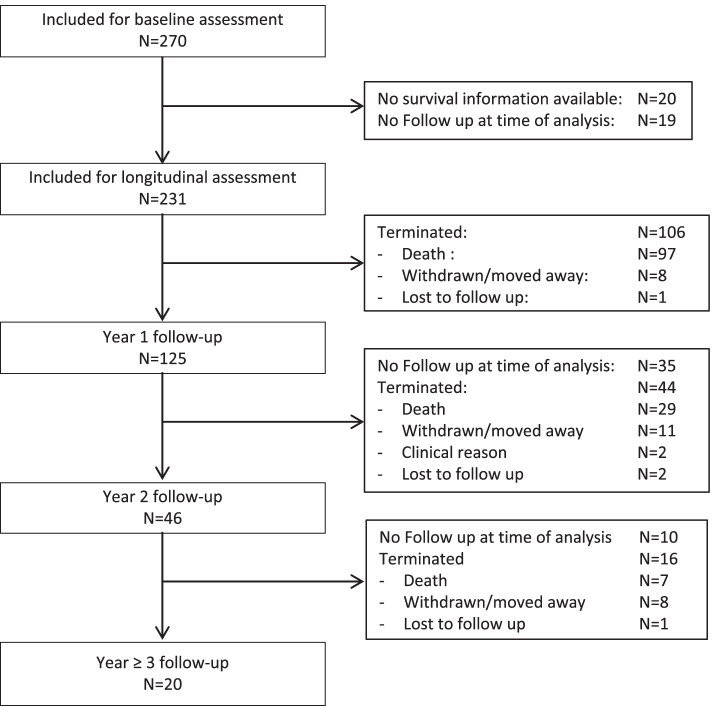
Participant flow. All women diagnosed with cervical cancer between 2013 and 2019 (*n* = 270) were included for baseline assessment. For those with at least one follow-up visit or available outcome tracing information (*n* = 231) longitudinal assessment was performed

Between June 2013 and September 2019, 270 out of 2078 screened women (44.9% HIV-positive) were diagnosed with CC and recruited into the study. Of those 123 (45.6%) were HIV infected and 147 (54.4%) HIV non-infected. Until September 2020 231 women (85.6%) had at least one follow-up visit or available outcome tracing information and could be therefore analyzed for survival assessments (Fig. 1). The median follow-up period was 11.9 months (range 0.2–67.2 months). One year after diagnosis, 97 (42%) and three years after diagnosis, 133 (57.6%) women had died. Overall, four participants were classified as LTFU (1.7%) and 27 (11.7%) had withdrawn their informed consent or relocated outside the Mbeya region. Beyond year 3, only eight women completed the fourth year, and two women the fifth year follow up visit**.**

Baseline characteristics and CC diagnostics for the overall study population and stratified by HIV status are shown in Table 1. In 98.9% of all cases CC diagnosis was confirmed by histology, 93% had a SCC and 5.9% an Adenocarcinoma. In three cases without histological confirmation the clinical presentation was highly suspicious for cancer.

**Table 1:** Baseline characteristics of HIV infected and non-infected women at the time of cervical cancer diagnosis

	**Total** **(*N* = 270)**	**HIV infected** **(*N* = 123)**	**HIV non-infected** **(*N* = 147)**
**Histology**			
Squamous Cell Carcinoma	251 (93)	117 (95.1)	134 (91.2)
Adenocarcinoma	16 (5.9)	4 (3.3)	12 (8.2)
No histology available	3 (1.1)	2 (1.6)	1 (0.7)
**Disease severity stage**			
Early stage I-IIA	28 (10.4)	16 (13.0)	12 (8.2)
Advanced stage IIB-IIIB	152 (56.3)	71 (57.7)	81 (55.1)
Late stage IV	5 (1.9)	1 (0.8)	4 (2.7)
Missing information	85 (31.5)	35 (28.5)	50 (34.0)
**Age at diagnosis in years, median (range)**	49.8 (21–87)	44.8 (21–76)	55.9 (31–87)
**Marital Status**			
Single	45 (1.9)	3 (2.6)	1 (0.7)
Married	147 (54.4)	55 (48.3)	87 (59.2)
Widowed	103 (38.2)	48 (42.1)	52 (35.4)
Divorced or separated	15 (5.6)	7 (6.1)	7 (4.8)
**Pregnancies & Birth**			
Gravidity, median (range)	6 (0–23)	4 (0–13)	7 (0–23)
Parity, median (range)	5 (0–20)	4 (0–12)	6 (0–20)
**Education**			
No education	106 (39.3)	27 (22)	79 (53.7)
Basic education	153 (56.7)	89 (72.4)	64 (43.5)
Advanced education	11 (4.1)	7 (5.7)	4 (2.7)
**Antiretroviral treatment (ART)**			
On ART		88 (71.6)	
-Years on ART, median (range)		4,5 (0–20)	
- < 1 year on ART		15 (12.2)	
- ≥ 1 year on ART		62 (50.4)	
Not on ART		23 (18.7)	
Not known		12 (9.8)	
**CD4 Counts**			
Recent CD4 count, median (range)		365 (11–1226)	
- ≤ 200 cells/µl		25 (20.3)	
-200–349 cells /µl		31 (25.2)	
- ≥ 350 cells /µl		68 (55.3)	
Lowest CD4 count, median (range)		231 (5–1158)	
- < 200 cells /µl		48 (39)	
CD4 cell count not known		5 (4.1)	
**WHO Stages**			
Stages 1–2		90 (73,2)	
Stages 3–4		19 (15.5)	
Not known		14 (11.4)	
**Clinical Presentation**			
Any symptoms	249 (92.2)	110 (89,4)	139 (94.6)
No symptoms	21 (7.8)	13 (10,6)	8 (5.4)
Lower abdominal pain	177 (65.6)	79 (64,2)	98 (66.7)
Abnormal vaginal discharge	223 (82.6)	96 (78.1)	127 (86.4)
Abnormal menstrual bleeding	62 (23)	34 (27.6)	28 (19.1)
-Intermenstrual bleeding	39 (14.4)	27 (22)	12 (8.2)
-Prolonged menstrual bleeding	29 (10.7)	10 (8.1)	19 (12.9)
Sexually active	*N* = 85	*N* = 46	*N* = 39
-Post-coital bleeding	60 (70.6)	34 (73.9)	26 (66.7)
-Pain during sex	70 (82.4)	38 (82.6)	32 (82.1)

**Fig. 2:**
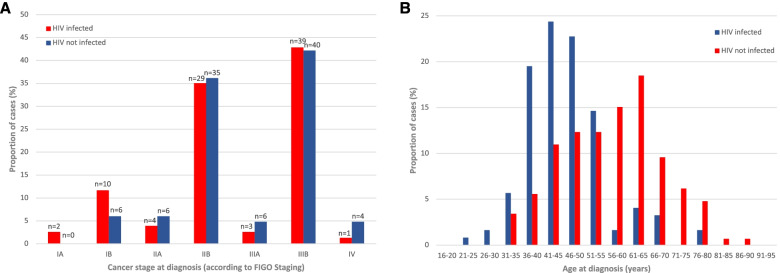
HIV infected and non-infected women according to age and stage of cancer disease. **A** Distribution, proportion, and number of cases by FIGO stage stratified by HIV infected and non-infected women. **B** Distribution and proportion of age groups at the time of cervical cancer diagnosis stratified by HIV infected and non-infected women. HIV infected women were 12 years younger than non-infected participants than HIV non-infected women (median age 44.8 vs. 56.4 years, *p* < 0.001). Despite this difference in age at diagnosis no difference in cancer stage at diagnosis was seen

Early-stage disease (FIGO I to IIA) was diagnosed in 10.4%, advanced stage (FIGO IIB to IIIB) in 56.3% and later stage (IV) in 1.9% of women, for the remaining 31.5% no staging was available. The distribution of FIGO staging for 185 women with available information was largely similar in HIV infected and non-infected women (Fig. 2A). The median age at cancer diagnosis was 49.8 years, and HIV infected women were at the time of diagnosis 12 years younger than HIV non-infected women (44.8 vs. 56.4 years, *p* < 0.001). The youngest age at cancer diagnosis in an HIV infected women was 21 years. Age group distribution at diagnosis is further shown in Fig. 2B, demonstrating the shift of diagnosis in HIV infected women towards the young with a peak in their 40 s, whereas HIV non-infected women peaked in their early 60 s. Women diagnosed before the age of 35 years were predominantly HIV infected.

Among HIV infected women, 88 (71.5%) were on ART at the time of cancer diagnosis, the median CD4-cell count was 365 cells/µl, and 20.3% had severe immune suppression < 200 cells/µL.

When asked for any symptoms possibly related to CC, abnormal vaginal discharge (82.6%) and lower abdominal pain (65.5%), and for sexually active women pain during sex (82.4%) and post coital bleeding (70.6%) were reported.

### Cancer care and treatment

**Fig. 3:**
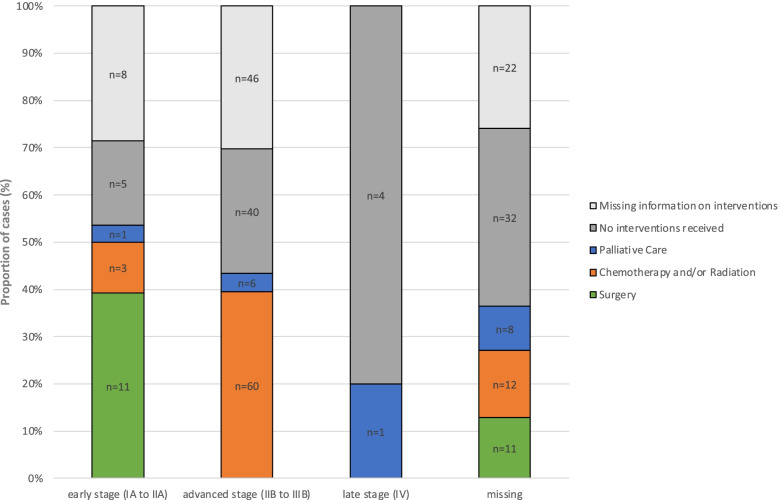
Numbers and proportions of participants receiving cancer-directed therapies (CDT) and palliative care by disease severity. Early-stage disease FIGO I to IIA, advanced stage disease FIGO IIB to IIIB, late-stage disease FIGO IV. No interventions indicate that participants did not receive or obtain any CDT nor palliative care as confirmed by tracing information

A referral letter that included clinical and pathological diagnostic results were provided to all women by the study clinic. Following linkage to the MZRH Gynecology and further ORCI in Dar es Salaam, 113 (42%) received medical treatment and care including surgery in 22 (8%) cases, radio-chemotherapy in 75 (28%) cases, or palliative care only in 16 cases (6%) mostly in the form of pain relieve. During tracing we obtained information about 81 women (30%) not receiving or requesting further care or treatment services, 44 of these women (54.3%) have had attended health care services after diagnosis for further treatment evaluation and counselling. In 76 cases (28%) no information on further treatment and care was available, but it can be assumed that most of these women also did not receive or request any further services, as many of those were in advanced stage disease (61% stage ≥ IIB) or died at home (19,7%). An overview of intervention by disease severity stage is provided in Fig. 3. Successful linkage to treatment and care services was significantly associated with women who were HIV co-infected (RR 1.35 (1.07–1.71), *p* = 0.012), or had higher level of education (RR 1.44 (1.10–1.89), *p* = 0.008). For other factors such as age (RR 1.00 (0.99–1.01), *p* = 0.573) or disease stage (RR 0.80 (0.60–1.08), *p* = 0.150) we did not find significant association with health care compliance. During home tracing visits, reasons for not sustained of rejected health care services attendance was asked from some of those women, however not systematically. Frequently provided answers included lack of trust in the medical care, the institutions and recommended interventions, fear of surgery, preferred options for spiritual healing, financial difficulties, lack of time with upcoming harvest seasons and overcrowded hospitals.

### Mortality and risk factors

Survival or mortality information was analyzed from 231 women for whom outcome information or follow-up visits were available. High mortality rates were characteristic in our study population with an overall median survival time of 15.2 months (IQR 7.3-not estimable) after diagnosis, and one- and three-year survival rates of 56.3% and 13.1%, respectively.

**Fig. 4:**
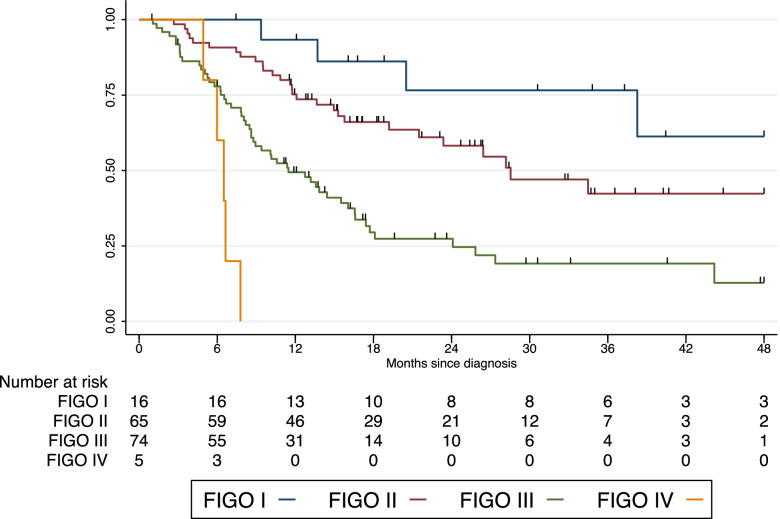
Kaplan Meier survival by FIGO stages. Significant lower survival estimates were seen with higher FIGO stages (*p* < 0.001)

Survival rates were significantly associated with FIGO stages as demonstrated by Kaplan Meier (Fig. 4) indicating statistically significant lower survival estimates with higher FIGO stages (median survival in FIGO stage I cancer more than 40 months, FIGO stage II 28.5 months, FIGO stage III 11.5 months and FIGO stage IV cancer 6.5 months, *p* < 0.001). Higher mortality rates were significantly associated with increasing FIGO stages (IRR 1.92 [95% CI 1.00–3.67], *p* = 0.049).

**Fig. 5:**
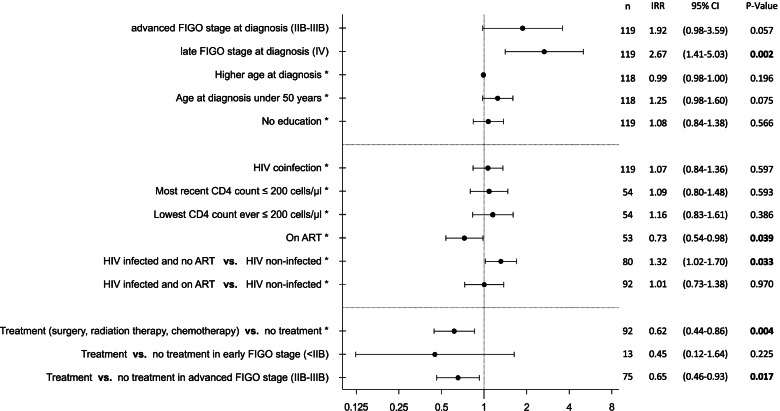
Factors associated with 2-year cancer mortality.Shown as incidence rate ratios on death (IRR), calculated by robust Poisson regression. *Adjusted by FIGO stage

Receiving CDT (surgery, radiation therapy, chemotherapy) was overall significantly associated with lower 2 years mortality rates (FIGO stage and age adjusted IRR 0.62 [95% CI 0.44–0.86], *p* = 0.004). This was significantly associated with advanced stage disease, but not significant with early-stage disease mortality, likely due to low sample size and consequently large confidence intervals (Fig. 5). Excluding women diagnosed in FIGO stage IV (none of them received CDT), the overall survival benefit was in median 27.7 months (median survival of women with no CDT 10.5 months [IQR 5.3–16.6 months] and versus 38.2 months [IQR 13.72– not estimable] with CDT, *p *< 0.001).

The HIV status did not significantly impact mortality rates (IRR 1.07, 95% CI 0.84–1.36; *p* = 0.597), with a median survival time of 17.7 months (IQR 7.8-not estimable) in HIV infected versus 13.7 months (IQR 6.5–38.3 months) in non-infected women (*p* = 0.108). In FIGO stage adjusted regression analysis a significant lower mortality rate was observed when comparing HIV-infected women on ART to women without current ART (IRR 0.73 [95% CI 0.54–0.98, *p* = 0.039]). HIV infected women not on ART also had a significant higher mortality rate compared to women with no HIV coinfection (IRR 1.32 (95% CI 1.02–1,70), *p* = 0.033), and when comparing HIV-infected women on ART versus not HIV infected women no difference in mortality was observed (Fig. 5). No significant change in rates of mortality was found for severe immune suppression as either expressed by most recent CD4 counts linked to the time of cancer diagnosis using 200 cells/µl as the cut-off (IRR 1.15 (95% CI 0.86–1.55), *p* = 0.349) or the lowest ever measured CD4 count using a cut-off of 200 cells/µl (IRR 1.07 (95% CI 0.80–1.44), *p* = 0.629).

No difference on mortality rates were seen in respect to age at cancer diagnosis or educational status. Overall, 58.7% of all women died at home, 39.9% of the women died in hospitals, the remaining 1.5% died in unknown other places.

## Discussion

The overall observation of this study was a high detection of women in advanced or late CC stage, both for HIV infected and non-infected women. This can be explained with the rather recent introduction of preventive cervical screening activities in our study region and late presentation at the clinics. Our study was not designed to provide representative population-based cancer prevalence information, as the study was targeting cancer cases. In addition, a majority of 92% of women reported symptoms possibly related to cancer diagnosis, which could indicate a sampling bias as symptomatic women preferentially might have attended screening services. However, our observations are in line with other cancer reports from Africa; a recent population-based registry study including 13 registries form 11 SAA countries accounted 65.8% of cases with stage III-IV disease [24]. Survival in our study was significantly associated with lower FIGO stages, and the 1 and 3 years survival rates were 56.3% and 13.1%, respectively – as compared to 69.8%, 44.5% and 33.1% at 1, 3 and 5 years from the African registry study [24].

All women in our study received medical counselling and a referral letter including histopathological results for further cancer care and treatment at the MZRH gynaecology department. For most participants (71.5%) we had information about successful linkage for further care and treatment from the gynaecology department, however, only 42% received cancer-directed therapy (CDT) or palliative support. This again is in line with a recently published register study including nine population-based cancer registries from eight SAA countries reporting that only 15.8% of CC patients received CDT with curative potential and in addition 22% without curative potential [25]. There are only few centers in Tanzania where CDT is available, and during the period of our study only the Ocean Road Cancer Institute (ORCI) in Dar Es Salaam provided radio-chemotherapy. In Tanzania cancer therapy is exempted from health care costs, but additional costs and logistical challenges such as for transport to cancer treatment centers or long-time accommodation at places away from home impose often not affordable burden to patients. In our study we included women from rural as well as urban areas, and especially women from rural areas were challenged in attending screening and health care services due to transportation, cost, and time barriers. In a study by ORCI numbers of attending patients from two referring areas in Tanzania, Mwanza and Mbeya region, were described and the authors concluded that a vast majority of CC patients did not received needed CDT, advocating the need to scale out decentralized cancer treatment facilities within the country [26].

Another barrier to cancer screening, prevention and CDT is lack of knowledge, attitude and practice towards offered or available health services, including fear, stigma and misinformation surrounding CC, which was recently reviewed for Tanzania [27]. In our study a substantial proportion of patients were not compliant to offered health services. Reported reasons following tracing information included lack of trust in the medical care, the institutions and recommended interventions, preferred options for spiritual or traditional healing, financial difficulties, lack of time with upcoming harvest seasons and overcrowded hospitals. Interestingly, successful linkage to treatment and care services in our study was significantly associated with higher education – which might have been biased as higher educated participants were more likely to come from the urban environment with shorter distances to the health facility. Another factor associated with successful linkage was HIV coinfection. It can be speculated that HIV infected women have a greater confidence and linkage to health care services because of their regular HIV care and treatment attendance. An important finding of our study for the local cancer treatment services was that CDT was significantly associated with a beneficial clinical impact expressed by greater survival time and lower 2-year mortality rates.

An important objective of our study was to investigate the impact of HIV co-infection on CC. We did not observe an impact of HIV co-infection on overall mortality rates or a trend towards disease severity as compared to not HIV infected patients. However, we found a significant impact of antiretroviral treatment resulting in comparable mortality rates for HIV-infected women on ART and HIV non-infected women. For HIV-infected women without ART higher mortality rates could be demonstrated in comparison to HIV-infected women on ART and HIV non-infected women. This might explain inconclusive results also reported in other studies. In a smaller study from Uganda the median survival for HIV infected women was significantly shorter than those of non-infected women, but when adjusted for FIGO stages Hazard ratios were not significantly different anymore [12]. A study from Botswana describes an remarkable adjusted doubled risk of death for HIV infected women, especially for women in early stages, which later on could not be confirmed by another analysis from Botswana seeing no difference in 2-year survival by HIV status [10, 28]. A study from South Africa was able to match an advanced staged cervical cancer cohort from 2007–2011, for whom HIV status was available, with a national population register to calculate a 5-year overall survival for all patients. They also observed a difference between HIV infected and non-infected women, but no difference between HIV infected women on ART and non-infected women [29].

An important finding in our study was that HIV infected women were significantly younger at the time of diagnosis. HIV infection is a risk factor for CC and it is estimated that infected women are six times more likely to get cervical cancer compared to women without HIV [9]. Infection with high risk HPV subtypes is the main factor for CC oncogenicity and HIV induced immunosuppression is associated with greater HPV infection rates and persisting HPV infection leading to cancer, thus leading to early onset of cancer [3, 6, 9]. In line with this we recently described in HIV-infected women within our 2H cohort an increased proportion of activated cervical T cells on the mucosal level, that together with an HIV-induced depletion of cervical CD4 T cells, may increase the risk for HPV infection, associated premalignant lesions and cancer in HIV + women [30]. CC screening guidelines by the WHO and Tanzanian National Guidelines therefore recommend screening services in HIV infected women and girls, who have initiated sexual activity regardless of age, when visiting the clinic for ART every six months [16, 18].

Limitations of this study include a possible sampling bias as indicated above as the study was targeting cancer cases and many women reported symptoms possibly related to cancer diagnosis that might have led to a greater screening services attendance. We also assume an operational study bias related to CDT compliance because of persistent effort to follow up on all women with CC diagnosis, including telephone visits and even physical home visits to rural areas. In contrast mortality or survival rates might be under- or overestimates as despite our tracing efforts follow-up information were often not obtainable.

In line with the call for global action made by the WHO to eliminate cervical cancer we aim to provide real-world data to assess the current cervical cancer care situation, as well as barriers and compliance in a resource poor-setting to support evidence based global health policies. Countries like Tanzania with up-scaling CC services are challenged by high numbers of diagnosed CC cases and sparse – however increasing—availabilities of decentralized CDT infrastructure providing cancer care and treatment. In combination with frequent late-stage presentation especially palliative care, e.g., symptom control currently mainly limited to pain relief, should be improved. CC education, reduction of stigmatization, fear, and improved patient-focused health service provision is needed to increase trust and compliance to the existing health care services. In our study we could demonstrate that CDT interventions have a clinical impact on survival also for remote settings. In combination with preventive cancer screening and HPV vaccination programmes, CDT imposes challenges on African health care systems, also in respect to health care system cost considerations as recently summarized in a WHO technical document for Tanzania [31]. Especially the scale-up of preventive HPV vaccination seems eminent, as quadrivalent HPV vaccination is associated with a substantially reduced risk of invasive cervical cancer at the population level as recently demonstrated [32]. To end the cervical cancer crisis the WHO recommends all countries should focus on the following three pillars: to get at least 90% of all girls fully vaccinated, 70% of all women screened, and 90% of all women with pre-cancer or invasive cancer treated or at least managed. Each country should meet the 90-70-90 targets by 2030 to get on the path to eliminate cervical cancer within the next century [33]. As in our study, long-time follow-up of real life cohorts are needed to inform African health systems about the implementation of services, challenges but also benefits including capacity building and knowledge transfer.

## Data Availability

The data of the 2H Study are available on different formats, and are electronically stored on a secured server. Access to data and data sharing is possible upon justified request to the corresponding author and is subject to 2H Study steering approval and data transfer agreement with the Tanzanian authorities. As the study was performed in close harmonization with the Tanzania Cervical Cancer Programme, we do not plan to publish the data on a publicly available depository to protect clinical sensitive programme data.

## References

[1] Fitzmaurice C, Allen C, Barber RM, Barregard L, Bhutta ZA, Global Burden of Disease Cancer C (2017). Global, Regional, and National Cancer Incidence, Mortality, Years of Life Lost, Years Lived With Disability, and Disability-Adjusted Life-years for 32 Cancer Groups, 1990 to 2015: A Systematic Analysis for the Global Burden of Disease Study. JAMA Oncol.

[2] Ferlay J, Ervik M LF, Colombet M, Mery L, Piñeros M, Znaor A, et al. Global Cancer Observatory: Cancer Today 2013 [updated December 2020. Available from: https://gco.iarc.fr/today.

[3] World Health Organization. Human papillomavirus (HPV) and cervical cancer 2020 [updated 2021. Available from: https://www.who.int/news-room/fact-sheets/detail/human-papillomavirus-(hpv)-and-cervical-cancer.

[4] Sung H, Ferlay J, Siegel RL, Laversanne M, Soerjomataram I, Jemal A (2021). Global cancer statistics 2020: GLOBOCAN estimates of incidence and mortality worldwide for 36 cancers in 185 countries. CA Cancer J Clin.

[5] de Sanjose S, Quint WG, Alemany L, Geraets DT, Klaustermeier JE, Lloveras B (2010). Human papillomavirus genotype attribution in invasive cervical cancer: a retrospective cross-sectional worldwide study. Lancet Oncol.

[6] Myers KO, Ahmed NU (2018). The Role of HIV in the Progression through the Stages of the Human Papillomavirus to Cervical Cancer Pathway. AIDS Rev.

[7] Chaturvedi AK, Madeleine MM, Biggar RJ, Engels EA (2009). Risk of human papillomavirus-associated cancers among persons with AIDS. J Natl Cancer Inst.

[8] Frisch M, Biggar RJ, Goedert JJ (2000). Human papillomavirus-associated cancers in patients with human immunodeficiency virus infection and acquired immunodeficiency syndrome. J Natl Cancer Inst.

[9] Stelzle D, Tanaka LF, Lee KK, Ibrahim Khalil A, Baussano I, Shah ASV (2021). Estimates of the global burden of cervical cancer associated with HIV. Lancet Glob Health.

[10] Dryden-Peterson S, Bvochora-Nsingo M, Suneja G, Efstathiou JA, Grover S, Chiyapo S (2016). HIV Infection and Survival Among Women With Cervical Cancer. J Clin Oncol.

[11] Coghill AE, Newcomb PA, Madeleine MM, Richardson BA, Mutyaba I, Okuku F (2013). Contribution of HIV infection to mortality among cancer patients in Uganda. AIDS (London, England).

[12] Wu ES, Urban RR, Krantz EM, Mugisha NM, Nakisige C, Schwartz SM (2020). The association between HIV infection and cervical cancer presentation and survival in Uganda. Gynecologic Oncol Rep.

[13] Mosha D, Mahande M, Ahaz J, Mosha M, Njau B, Kitali N (2009). Factors associated with management of cervical cancer patients at KCMC Hospital, Tanzania: a retrospective cross-sectional study. Tanzan J Health Res.

[14] Kelly H, Weiss HA, Benavente Y, de Sanjose S, Mayaud P, Art (2018). Association of antiretroviral therapy with high-risk human papillomavirus, cervical intraepithelial neoplasia, and invasive cervical cancer in women living with HIV: a systematic review and meta-analysis. Lancet HIV..

[15] Peirson L, Fitzpatrick-Lewis D, Ciliska D, Warren R (2013). Screening for cervical cancer: a systematic review and meta-analysis. Syst Rev.

[16] The United Republic of Tanzania Ministry of Health (2011). Tanzania Service Delivery Guidelines for Cervical Cancer Prevention and Control.

[17] The United Republic of Tanzania Ministry of Health (2016). One Plan II: The National Road Map Strategic Plan To Improve Reproductive, Maternal, Newborn, Child & Adolescent Health in Tanzania (2016–2020).

[18] World Health Organization (2014). Comprehensive Cervical Cancer Control: A guide to essential practice - 2nd ed.

[19] Vogel UF. Towards universal access to prevention, treatment and care: experiences and challenges from the Mbeya Region in Tanzania—a case study. Geneva: UNAIDS; 2007. https://www.unaids.org/sites/default/files/media_asset/jc1291_mbeya_en_0.pdf..

[20] McHaro R, Lennemann T, France J, Torres L, Gari M, Mbuya W (2021). HPV Type Distribution in HIV Positive and Negative Women With or Without Cervical Dysplasia or Cancer in East Africa. Front Oncol.

[21] Solomon D, Davey D, Kurman R, Moriarty A, O’Connor D, Prey M, et al. The 2001 Bethesda System: terminology for reporting results of cervical cytology. JAMA. 2002;287(16):2114–9. 10.1001/jama.287.16.2114 11966386

[22] Pecorelli S (2009). Revised FIGO staging for carcinoma of the vulva, cervix, and endometrium. Int J Gynaecol Obstet.

[23] Piver MS, Rutledge F, Smith JP (1974). Five classes of extended hysterectomy for women with cervical cancer. Obstet Gynecol.

[24] Sengayi-Muchengeti M, Joko-Fru WY, Miranda-Filho A, Egue M, Akele-Akpo MT, N’da G (2020). Cervical cancer survival in sub-Saharan Africa by age, stage at diagnosis and Human Development Index: A population-based registry study. Int J Cancer.

[25] Griesel M, Seraphin TP, Mezger NCS, Hammerl L, Feuchtner J, Joko-Fru WY (2021). Cervical Cancer in Sub-Saharan Africa: A Multinational Population-Based Cohort Study of Care and Guideline Adherence. Oncologist.

[26] Gesink MP, Chamberlain RM, Mwaiselage J, Kahesa C, Jackson K, Mueller W (2020). Quantifying the under-estimation of cervical Cancer in remote regions of Tanzania. BMC Cancer.

[27] Runge AS, Bernstein ME, Lucas AN, Tewari KS (2019). Cervical cancer in Tanzania: A systematic review of current challenges in six domains. Gynecologic Oncology Reports.

[28] Grover S, Bvochora-Nsingo M, Yeager A, Chiyapo S, Bhatia R, MacDuffie E (2018). Impact of Human Immunodeficiency Virus Infection on Survival and Acute Toxicities From Chemoradiation Therapy for Cervical Cancer Patients in a Limited-Resource Setting. Int J Radiat Oncol Biol Phys.

[29] Simonds HM, Botha MH, Neugut AI, Van Der Merwe FH, Jacobson JS (2018). Five-year overall survival following chemoradiation among HIV-positive and HIV-negative patients with locally advanced cervical carcinoma in a South African cohort. Gynecol Oncol.

[30] Mbuya W, McHaro R, Mhizde J, Mnkai J, Mahenge A, Mwakatima M (2020). Depletion and activation of mucosal CD4 T cells in HIV infected women with HPV-associated lesions of the cervix uteri. PLoS ONE.

[31] World Health Organization (2020). Costing the National Response to Cervical Cancer: United Republic of Tanzania, 2020–2024.

[32] Lei J, Ploner A, Elfstrom KM, Wang J, Roth A, Fang F (2020). HPV Vaccination and the Risk of Invasive Cervical Cancer. N Engl J Med.

[33] World Health Organization (2020). Global strategy to accelerate the elimination of cervical cancer as a public health problem.

